# Supplementary material for: Detecting the effect of genetic diversity on brain composition in an Alzheimer’s disease mouse model
Source: Commun Biol. 2024 May 20;7:605. doi: 10.1038/s42003-024-06242-1 (PMC11106287; doi:10.1038/s42003-024-06242-1)
Supplement: Supplementary file 2 — Supplementary Information [file 42003_2024_6242_MOESM2_ESM.pdf]

Supplemental Figure 1

a

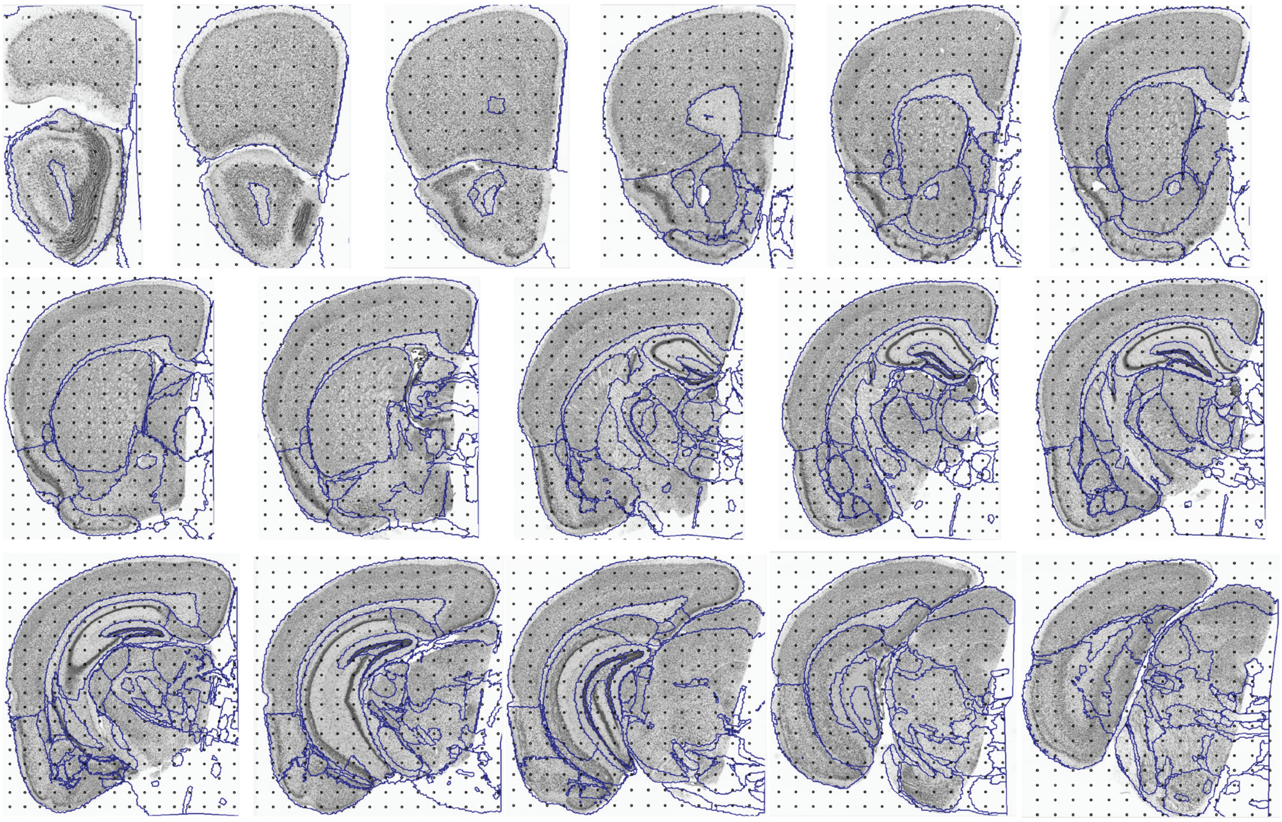

b

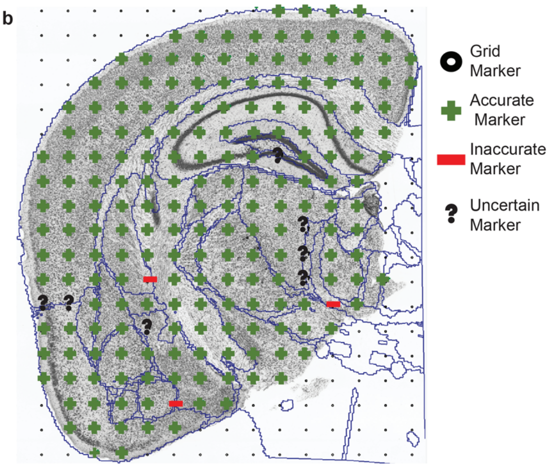

Supplemental Figure 1. Intermediate hierarchy and QCAlign quality control assessment of atlas registration of thionine sections. a.) Intermediate hierarchy depiction over every thionine section of a representative brain following atlas registration using QuickNII and VisuAlign. Allen Mouse Brain Atlas CCFv3 regions were compiled to make an intermediate hierarchy that promotes the assessment of regional registration. b.) Representative quality control assessment of the atlas registration of a thionine slice in QCAlign. Raters assigned grid markers verifying the registration of each point as either accurate, inaccurate, or uncertain.

Supplemental Figure 2

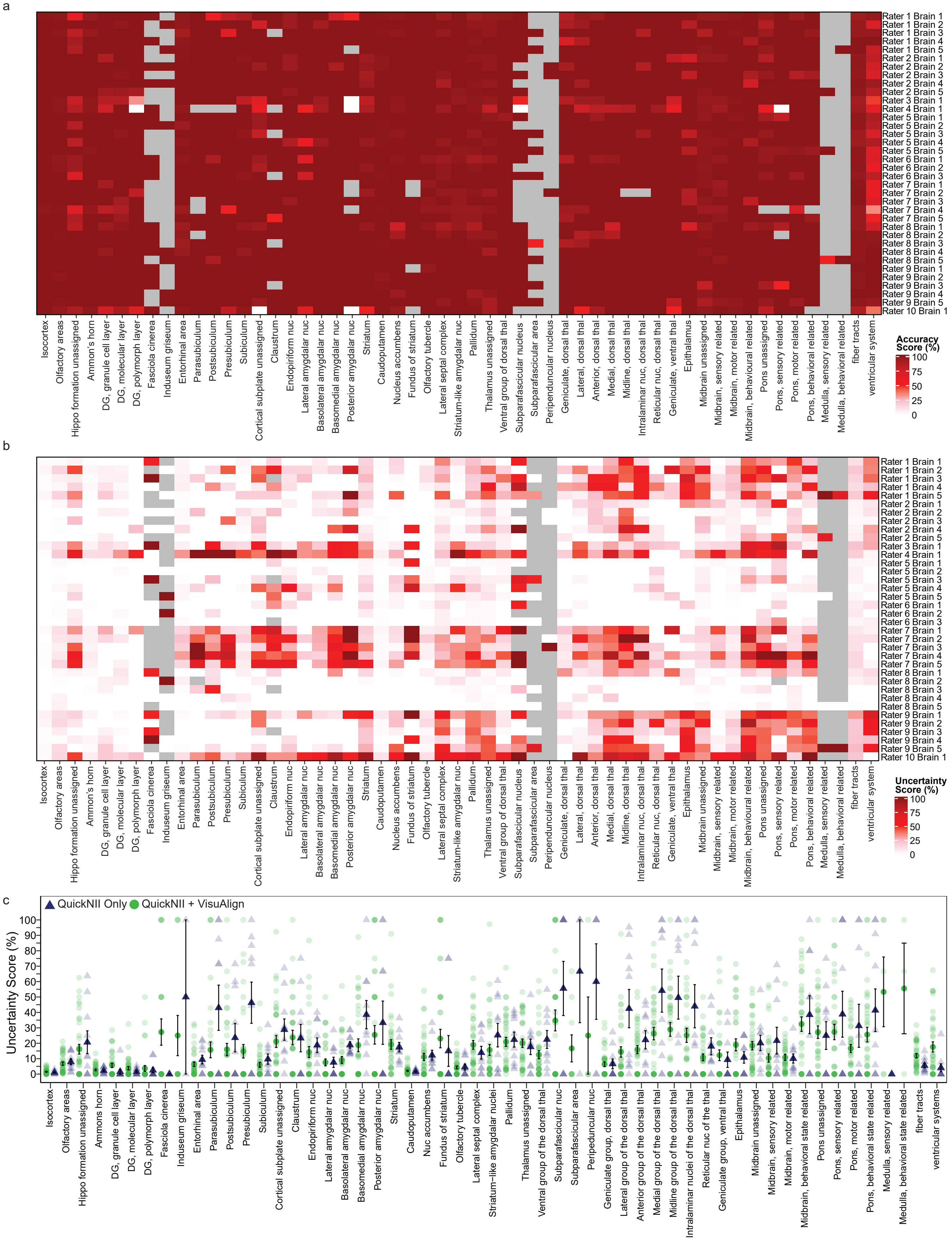

Supplemental Figure 2: QCAlign scores achieved via quality control assessment of intermediate hierarchy regions. a.) Heatmap of regional accuracy scores per rater per brain. b). Heatmap of regional uncertainty scores per rater per brain. Gray regions were not represented in the brain series and/or did not receive QCAlign scores for the measure. c.) Mean uncertainty scores per intermediate hierarchy region after QuickNII registration alone (navy triangles) or after QuickNII and VisuAlign registration (green circles). Two raters scored the same 5 randomly selected brains after QuickNII registration alone, max n=10 assessments per region (Raters: n= 2 per brain). Up to 10 raters scored the same 5 randomly selected brains after QuickNII and VisuAlign registration, max n=36 assessments per region (Raters: n= 6-10 per brain, see Supplemental Data 2 for the exact number of assessments measured per region and registration method). Dark shapes represent the mean score across raters per region for 5 brains +/- SEM, with the opaque shapes representing the individual assessments contributing to each mean calculation.

Supplemental Figure 3

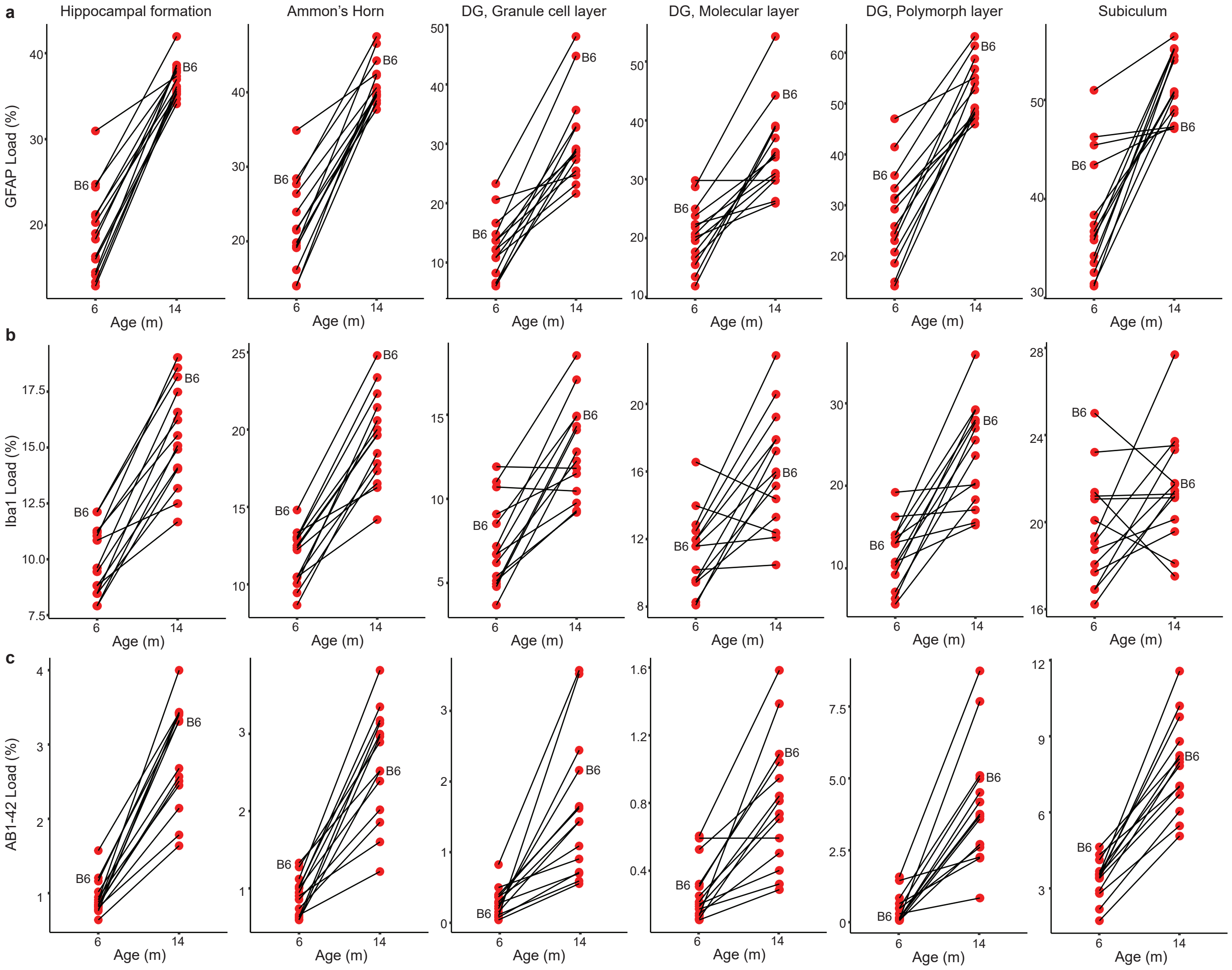

Supplemental Figure 3. Variation in stain load exists among AD-BXD strains.
Strain averages of a.) GFAP, b.) Iba1, and c.) AB1-42 load across the hippocampal formation and hippocampal intermediate hierarchy subregions. Points are mean load per strain. Each line connects a pair of strain averages across the age groups: 6m and 14m. Only strains with an aged match counterpart are represented (5XFAD mice only, n=14 strains per age group, n= 1-3 biological replicates per strain, 6m: n=17 mice (n=1 mouse/strain (B6 x BXD100, BXD44, BXD51, BXD60, BXD61, BXD62, BXD65, BXD69, BXD75, BXD77, BXD87, and C57BL/6J strains), n= 2 mice/strain (B6 x BXD32), n= 3 mice/strain (B6 x DBA/2J)), 14m: n=18 mice (n=1 mouse/strain (B6 x BXD100, BXD44, BXD51, BXD60, BXD61, BXD62, BXD69, BXD75, BXD77, BXD87, and C57BL/6J), n= 2 mice/strain (B6 x BXD32 and BXD65), n= 3 mice/strain (B6 x DBA/2J))). The B6 founder strain is labeled for reference.

FileName	Harvest ID	Strain	Sex	Harvest Age (m)	5XFAD Genotype	Hemisphere	Bulk RNAseq Analyzed
05-12-15-36	05-12-2015.36	B6 x BXD32	Male	6	5XFAD	Right	Yes
06-04-15-04	06-04-2015.04	B6 x BXD32	Female	6	5XFAD	Left	Yes
12-14-15-19	12-14-2015.19	B6 x BXD32	Female	14	5XFAD	Left	Yes
12-18-15-08	12-18-2015.08	B6 x BXD32	Male	14	5XFAD	Left	Yes
06-24-15-06	06-24-2015.06	B6 x BXD44	Female	6	5XFAD	Left	Yes
09-23-15-08	09-02-2015.08	B6 x BXD44	Female	6	NTG	Left	No
12-09-15-12	12-09-2015.12	B6 x BXD44	Female	14	5XFAD	Left	Yes
03-14-16-03	03-14-2016.03	B6 x BXD51	Female	14	5XFAD	Left	Yes
09-02-15-04	09-02-2015.04	B6 x BXD51	Female	6	5XFAD	Right	Yes
05-12-15-27	05-12-2015.27	B6 x BXD60	Female	6	5XFAD	Right	No
11-15-15-05	11-16-2015.05	B6 x BXD60	Female	14	5XFAD	Left	Yes
04-26-15-06	04-26-2015.06	B6 x BXD61	Female	6	5XFAD	Left	Yes
12-07-15-08	12-07-2015.08	B6 x BXD61	Female	14	5XFAD	Right	Yes
05-22-15-10	05-22-2015.10	B6 x BXD62	Female	6	5XFAD	Right	Yes
12-02-15-14	12-02-2015.14	B6 x BXD62	Female	14	5XFAD	Right	Yes
03-28-16-10	03-28-2016.10	B6 x BXD65	Female	14	5XFAD	Left	No
03-28-16-18	03-28-2016.18	B6 x BXD65	Female	14	5XFAD	Right	Yes
11-23-15-03	11-23-2015.03	B6 x BXD65	Female	6	5XFAD	Right	Yes
04-26-15-17	04-26-2015.17	B6 x BXD66	Female	6	NTG	Left	No
12-14-15-04	12-14-2015.04	B6 x BXD66	Female	14	5XFAD	Right	Yes
01-11-16-12	01-11-2016.12	B6 x BXD68	Female	14	5XFAD	Left	Yes
09-23-15-04	09-23-2015.04	B6 x BXD68	Female	6	NTG	Right	No
05-12-15-41	05-12-2015.41	B6 x BXD69	Female	6	5XFAD	Right	Yes
11-09-15-18	11-09-2015.18	B6 x BXD69	Female	14	5XFAD	Left	Yes
01-20-16-17	01-20-2016.17	B6 x BXD75	Female	14	5XFAD	Right	Yes
06-24-15-13	06-24-2015.13	B6 x BXD75	Female	6	5XFAD	Left	Yes
05-12-15-05	05-12-2015.05	B6 x BXD77	Female	6	5XFAD	Right	Yes
11-09-15-16	11-09-2015.16	B6 x BXD77	Female	14	5XFAD	Left	Yes
01-18-16-16	01-18-2016.14	B6 x BXD87	Female	14	5XFAD	Right	Yes
06-24-15-08	06-24-2015.08	B6 x BXD87	Female	6	5XFAD	Right	Yes
05-12-15_02	05-12-15.02	B6 x BXD10(	Female	6	5XFAD	Right	No
11-16-15-09	11-16-2015.09	B6 x BXD10(	Female	14	5XFAD	Left	Yes
09-23-15-06	09-23-2015.06	B6 x DBA/2J	Female	6	5XFAD	Right	Yes
10-14-15-07	10-14-2015.07	B6 x DBA/2J	Female	14	5XFAD	Left	Yes
11-15-15-13	11-16-2015.13	B6 x DBA/2J	Female	14	5XFAD	Left	Yes
11-2014-01	11-00-2014.01	B6 x DBA/2J	Female	6	5XFAD	Left	Yes
11-2015-02	11-00-2014.02	B6 x DBA/2J	Female	6	5XFAD	Left	Yes
11-23-15-17	11-23-2015.17	B6 x DBA/2J	Female	14	5XFAD	Right	Yes
10-14-15-14	10-14-2015.14	C57BL/6J	Female	14	5XFAD	Left	Yes
11-09-15-20	11-09-2015.20	C57BL/6J	Female	6	5XFAD	Left	Yes

Supplemental Table 1. Strain, sex, age, 5XFAD genotype, hemisphere, and RNAseq inclusion metadata for all animals with IHC completed for this study.

Primary	Dilution	Secondary	Dilution
Aβ1-42, Biolegend 825301, Host: Rabbit	1:12500	Anti-rabbit biotinylated, Vector BA-2001, Host: Goat	1:1000
GFAP, Dako Z0334, Host: Rabbit	1: 14000	anti-rabbit, Vector BA-1000, Host: Goat	1:1000
Iba1, Wako 019-19741, Host: Rabbit	1:6000	anti-rabbit, Vector BA-1000, Host: Goat	1:1000
anti-NeuN antibody, biotin conjugated, Millipore MAB377B, Host: Rabbit	1:1500	N/A (Primary Ab is Biotinylated)	N/A
Thionine Nissl, Source: Fisher, Catalog #: T40925	0.05% thionine/acetate [illegible]	N/A	N/A

Supplemental Table 2. Antibody and dilution information used by NSA for IHC staining.

Brain ID	Brain Sections Removed From Nutil Analysis		
1-11-16_12			
1-18-16_16	NeuN-1-18-16_16-09		
1-20-16_17	NeuN-1-20-16_17-03		
03-14-16_03	Thionine-03-14-16-03-05	NeuN-03-14-16_03-19	
03-28-16_10			
03-28-16_18			
04-26-15_06			
04-26-15_17	NeuN-04-26-15_17-18	Thionine-04-26-15-17-18	
5-12-15_02	GFAP-5-12-15-02-08	Thionine-5-12-15-02-18	
5-12-15_05			
5-12-15_27	AB1-42-5-12-15_27-06		
5-12-15_36			
5-12-15_41	Iba1-5-12-15-41-08		
5-22-15_10	NeuN-5-22-15_10-19	AB1-42-5-22-15_10-10	Iba1-5-22-15-10-10
06-24-15_06			
06-24-15_08			
06-24-15_13			
6-4-15_04			
09-23-15_04			
9-02-15_04			
9-23-15_06			
10-14-15_07			
10-14-15_14	NeuN-10-14-15_14-04		
11-09-15_16	Iba1-11-09-15-16-02	AB1-42-11-09-15_16-10	Iba1-11-09-15-16-10
11-09-15_18	AB1-42-11-09-15_18-10	GFAP-11-09-15-18-10	Iba1-11-09-15-18-10
11-09-15_20	Thionine-11-09-15-20-18		
11-15-15_05			
11-15-15_13			
11-16-15_09			
11-23-15_03			
11-23-15_17			
11-2014_01			
11-2015_02	Thionine-11-2015-02-13		
12-02-15_14			
12-07-15_08			
12-09-15_12	Thionine-12-09-15-12-05		
12-14-15_04			
12-14-15_19	NeuN-12-14-15_19-19		
12-18-15_08			

Supplemental Table 3. List of sections removed from individual stain and brain Nutil quantification. Listed sections include those that had greater than 30% damage as measured in QCAlign or were excluded following manual inspection indicating that the majority of the section was distorted and unfit for quantification.
