# Supplementary material for: Detecting the effect of genetic diversity on brain composition in an Alzheimer’s disease mouse model
Source: Commun Biol. 2024 May 20;7:605. doi: 10.1038/s42003-024-06242-1 (PMC11106287; doi:10.1038/s42003-024-06242-1)
Supplement: Supplementary file 3 — Description of Additional Supplementary Files [file 42003_2024_6242_MOESM3_ESM.pdf]

Description of Additional Supplementary Files

File name: Supplemental Data 1

Description: Customized regions input into the Nutil software. This list consists of the 77 intermediate hierarchy regions established in QCAlign for the study (columns B- BZ), in addition to the default regions provided in Nutil (columns CA – CF). The Allen Mouse Brain Atlas CCFv3 region IDs from which each customized region is compiled are listed vertically.

File name: Supplemental Data 2

Description: Number of QCAlign assessments compiled per region per registration method. For the registration achieved with QuickNII only, a maximum of 10 assessments were averaged across 2 raters and 5 brains. For the registration achieved with QuickNII and VisuAlign, a maximum of 36 assessments were averaged across 6-10 raters and 5 brains. The number of QCAlign assessments contributing to the mean accuracy and uncertainty scores are listed per region and registration method.

File name: Supplemental Data 3.

Description: Wilcoxon test results assessing the difference in stain load quantified following QuickNII registration alone or after QuickNII and VisuAlign registration for the 55 regions assessed in Figure 3b. The regional load per stain per age group among 5XFAD animals was compared between the two methods.

File name: Supplemental Data 4.

Description: ANOVA results comparing regional stain load for all intermediate hierarchy regions between 6m and 14m 5XFAD animals. FDR-corrected pvalues are indicated as FDR_adjusted_pval.

File name: Supplemental Data 5.

Description: Multilevel correlation results comparing gene expression and hippocampal load correlations both before and after age adjustment for 34 5XFAD animals. FDR-corrected p-values are indicated as Age/Non-adjusted p-value (FDR corrected).

File name: Supplemental Data 6.

Description: Post-analysis region exclusion parameters. List of 77 regions (compiled by QCAlign from CCFv3 regions) and 5 additional summary regions (Nutil provided default regions, also compiled from CCFv3 regions) organized by their inclusion or exclusion from QCAlign analysis as represented in Figure 3, Supplemental Figure 2, Nutil analysis as represented in figure 4a, or IHC and RNAseq integration in figures 5 and 6. "Parent term" are parent IDs, which do not represent any pixels in the CCFv3 and therefore did not generate results; "unassigned pixels" are pixels that are not assigned to a subregion but are instead labeled according to the parent region to which they belong within the Allen Mouse Brain Atlas CCFv3 2015; "low sampling" indicates that less than 20 assessments out of 36 total possible assessments contributed to the mean accuracy QCAlign score for these regions following QuickNII and VisuAlign registration. Some regions were excluded as they had been removed from the brain prior to IHC.
